# RANDOMIZED EXPERIMENTAL STUDY OF TOPICAL VASODILATORS IN MICROSURGERY WITH COST ANALYSIS

**DOI:** 10.1590/1413-785220243203e276513

**Published:** 2024-07-22

**Authors:** Renato Polese Rusig, Debora Yumi Yoshimura Orlandin Alves, Amanda de Oliveira Silva Nascimento, Gustavo Bispo dos Santos, Rames Mattar, Raquel Bernardelli Iamaguchi

**Affiliations:** 1. Universidade de São Paulo, Faculdade de Medicina, Hand Surgery and Reconstructive Microsurgery Group, Instituto de Ortopedia e Traumatologia Hospital das Clínicas HC-FMUSP, São Paulo, SP, Brazil.

**Keywords:** Microsurgery, Vasodilator Agents, Animal Models, Experimental Study, Complications, Microcirurgia, Complicações, Dilatação, Eficácia, Custo e Análises de Custo

## Abstract

**Objective:**

Throughout microsurgical anastomosis, many surgeons use topical vasodilators in order to reduce pathological vasospasm. It was carried out an experimental study comparing the effectiveness of topical use of Nitroglycerin, Papaverine, Magnesium sulfate over a control group in the femoral artery and vein of rats, in reducing prolonged vasospasm.

**Methods:**

Randomized comparative experimental study in 15 rats, divided into four groups. The external diameter of the vases soaked in the randomized solution was measured. For statistical analysis, it was calculated the percentual increase in the external diameter of the vessels.

**Results:**

A statistically significant increase in arterial dilation was observed after 10 minutes of topical application of 10% magnesium sulfate compared to the control group, with *p = 0.044* . No other drug showed a vasodilator effect superior to the control group. Magnesium sulfate at 10% is still not used in microsurgery and costs 15 times less than papaverine, the standard drug for topical vasodilation in clinical cases at our service.

**Conclusion:**

Magnesium sulfate had better vasodilating effects over the control group after 10 minutes of arterial microanastomosis. None of the tested drugs have presented superior vasodilating effects over each other nor the control group after venous microanastomosis. *Level of evidence II, Experimental study, Randomized Trial.*

## RESUMO

**Objetivo:**

Durante a anastomose microcirúrgica, muitos cirurgiões utilizam vasodilatadores tópicos para reduzir o vasoespasmo prolongado patológico, assim reduzindo o risco de complicações vasculares. Entretanto, ainda faltam dados experimentais para identificação da droga padrão-ouro para vasodilatadores tópicos em microcirurgia e sua avaliação de análise de custo, já que a droga geralmente utilizada para este objetivo é baseada, na maior parte dos casos, na experiência do cirurgião.

**Métodos:**

Foi realizado um estudo experimental comparativo randomizado, avaliando a eficácia do uso tópico de Nitroglicerina, Papaverina e Sulfato de Magnésio em relação a um grupo controle, na redução do vasoespasmo na artéria e veia femoral de ratos. Foram avaliados o diâmetro externo dos vasos embebidos em solução randomizada dos fármacos para vasodilatação. Após cálculo do aumento percentual no diâmetro externo dos vasos, foi realizada análise estatística.

**Resultados:**

Observou-se aumento estatisticamente significativo da dilatação arterial após 10 minutos de aplicação tópica de sulfato de magnésio a 10% em relação ao grupo controle, com p = 0,044. Nenhuma outra droga apresentou efeito vasodilatador superior ao grupo controle. O sulfato de magnésio a 10% ainda não é utilizado em microcirurgia e apresenta custo até 15 vezes menor quando comparado com a papaverina, droga padrão para vasodilatação tópica em casos clínicos em nosso serviço.

**Conclusão:**

O sulfato de magnésio apresentou melhor efeito vasodilatador quando comparado ao grupo controle, após 10 minutos da microanastomose arterial. Nenhum dos fármacos testados apresentou efeito vasodilatador superior após a microanastomose venosa. Nível de Evidência II, Estudo experimental, Ensaio Randomizado.

## INTRODUCTION

 Microsurgical free flaps currently have a high success rate in reconstructive surgeries, but complications may be present in 5-10% of cases. ^1^ One of the main causes of complications is vascular thrombosis, which can be caused by prolonged vasospasm of the vascular pedicle ^2^ ^-^ ^4^ due to a significant reduction in flow velocity. Decreased intraluminal flow promotes the formation of intraoperative thrombi, reduced blood supply to the tissue to be transferred and consequently: flap loss due to ischemia, ischemia-reperfusion injury, microcirculation thrombosis and altered consumption of coagulation factors. ^4^ ^,^ ^5^ ^,^ ^6^

 The use of vasodilators to control vasospasm in microsurgery is widely adopted; however, there is no drug defined as the gold standard for topical or systemic intraoperative use. ^7^ ^,^ ^8^ In current microsurgery, vasospasm is treated according to personal or institutional experience, there are few studies comparing the available drugs and the vasodilating potential of each drug. ^7^ ^,^ ^8^ ^,^ ^9^

 The objective of this experimental study is to find which topical vasodilating drug would be the most effective and least costly for use in microsurgery, including the effect on arterial and venous anastomoses. The following topical vasodilators were compared with the control group with physiological solution: papaverine, currently used as the medication of choice in our service; 10% magnesium sulfate, as it is a vasodilator with good efficacy in experimental studies and with antithrombotic effect by decreasing platelet aggregation; ^10^ ^,^ ^11^ and nitroglycerin, a drug widely used in cardiac surgery as an arterial vasodilator. ^12^ ^,^ ^13^ Quantitative effects were analyzed with the topical use of each drug in the femoral artery and vein of rats by measuring the external diameter of the vessels with a micrometer ruler, before and after microanastomosis. The secondary objective was to provide cost analysis of the drugs tested and availability in the context of Brazilian public health care, for intraoperative use in clinical occurrence. 

## MATERIAL AND METHOD

Fifteen male Wistar rats weighing 376–432 grams were included in this study. The study was submitted to and approved by the Animal Research Ethics Committee (CEUA) number 1752/2022 and CAAE number 29539719.8.0000.0068. The rats were kept in the vivarium and in a musculoskeletal research laboratory according to the principles of care and use of animals in laboratory.

 The animals were submitted to pre-anesthetic medication using tramadol hydrochloride at a dose of 0.3 mg/100 g associated with meloxicam at a dose of 0.02 mg/100 g intramuscularly and placed in an inhalational anesthetic induction box with Isoflurane (equipamento Bonther, Ribeirão Preto - SP). After confirmation of the anesthetic plan, the animals were placed in a nebulization mask with 2% Isoflurane, with bilateral trichotomy of the inguinal region and the specimen was positioned in horizontal dorsal decubitus on a surgical platform under a surgical microscope with magnification of up to 20x. The experimental tests were performed by the first author, under supervision and monitoring for the measurements and annotations of the data by the other authors. Surgery was always initiated on the right side with oblique inguinal incision, cauterization of vascular branches and microscopic dissection of the femoral artery, nerve and vein bilaterally, in its middle third. Transverse arteriotomy and microanastomosis were performed, followed by transverse venotomy and microanastomosis. Using a 10 mm optical glass eye ruler with a 100 micrometer scale, the external diameter of the femoral artery was measured distally to the microanastomosis, in favor of arterial flow, and the external diameter of the femoral vein was measured proximally to the microanastomosis, in favor of venous flow, and the values were recorded as time zero (t _0_ ), under magnification of 20x. We continued with topical application of vasodilators, all at room temperature, according to the following groups: 

Group 1: Nitroglycerin (50 mg/mL ampoule diluted in 0.9% sodium chloride to 0.4 mg/mL concentration - Tridil® Cristália Químico, Itapira - SP)

Group 2: Papaverine (50 mg/mL ampoule diluted in 0.9% sodium chloride to 30 mg/mL concentration - Hypoverin® Hypofarma, Ribeirão das Neves - MG)

Group 3: Magnesium sulfate (10% ampoule used at 100 mg/mL concentration - Samtec Biotecnologia Limitada, Ribeirão Preto - SP)

Group 4 (Control): 0.9 % sodium chloride

**Figure 1.**
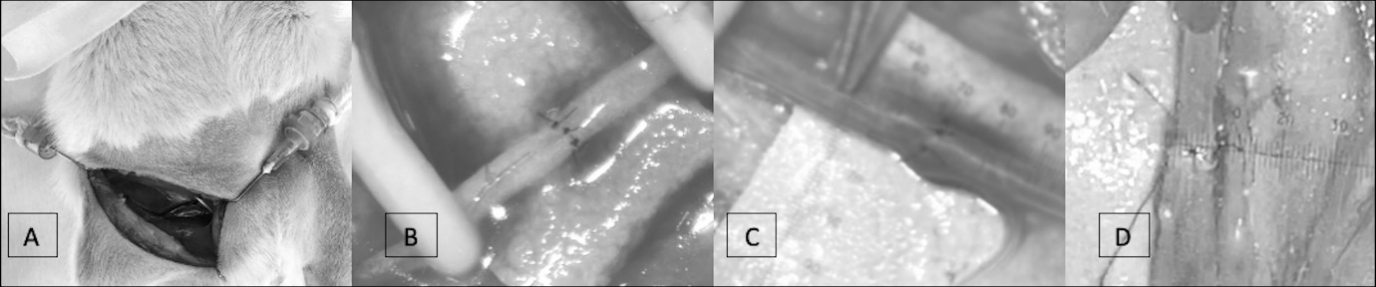
Demonstration of the stages of the experimental study. A: Surgical access of the inguinal region of the rat; B: Microanastomosis with the aid of Clamps; C: Measurement at initial time; D: Measurements after topical application of the drug.

 According to the groups described, we performed topical application of 4 ml of one of the solutions immediately after the measurement at the initial time (t _0_ ), in a order that was randomized through a platform ( https://www.random.org/ ) from 1 to 30, with the odd numbers referring to the right side and the even numbers referring to the left side. The vessels were kept soaked in the solution for 30 minutes and the external diameter of the femoral arteries and veins was measured with 3 min (t _3_ ), 5 min (t _5_ ), 10 min (t _10_ ) and 30 min (t _30_ ) counted from the application of the drugs (t _0_ ). Concomitantly with the measurement of the diameter of the vessels on the right side, the same procedure was performed on the left side and the measurements were recorded. Soon after the experimental phase described, the animals were euthanized using the anesthesia protocol and, after further confirmation of the anesthetic plan, 20% potassium chloride was administered intracardially. Figure 1

 Quantitative analysis of artery vasodilation was performed separately from the vein, following the same protocol for both: we calculated the difference in the external diameter of the artery (Δ _artery_ ) and vein (Δ _vein_ ) at the evaluated times (t _3_ , t _5_ , t _10_ and t _30_ ) in relation to the initial measurement (t _0_ ) [Δ _t_ = x _t_ - x _t0_ ], just before administering the drug, and their representation in relation to t _0_ expressed as a percentage. For statistical analysis, the SPSS version 20.0 program (SPSS Inc ®, Chicago, IL, USA) was used, with descriptive statistics and statistical analysis. After Levene’s test, the Kruskal Wallis test was used for quantitative variables, being considered statistically significant *p < 0.05* . After identifying a statistically significant result, the Mann-Whitney test was used to determine which drug analyzed produced greater vasodilation compared to the different drug groups and in relation to the control group, for the artery or for the vein. 

 The cost of papaverine, nitroglycerin and magnesium sulfate was researched in the regulations of the National Health Surveillance Agency (ANVISA) on the price of medicines defined by the Chamber of Regulation of the Drug Market (CMED) published between August 2022 and January 2023. ^14^ The factory price (FP) with Tax on Circulation of Goods and Services (ICMS) at 18% of each drug was used as a reference for comparative purposes. ^15^

## RESULTS

 We performed 26 tests on 15 rats. We obtained Group 1: Nitroglycerin n = 6, Group 2: Papaverine n = 7, Group 3: Magnesium Sulfate n = 7, Group 4: Control n = 6. The mean values of the external diameter of the arteries and veins are exemplified in Tables 1 and 2 , respectively. Four cases were excluded due to cardiorespiratory arrest of the rat during the procedure, before the measurement at 10 minutes (3 cases in the Papaverine group and 1 case in the Nitroglycerin group). 

**Table 1.** Mean arterial external diameter measurements, in micrometers (µm) according to the time (t) evaluated after drug administration.

Group	Drug	Time									
		t _0_		t _3_		t _5_		t _10_		t _30_	
		Mean	SD	Mean	SD	Mean	SD	Mean	SD	Mean	SD
1	Nitroglycerin	7.40	1.14	8.20	0.84	8.20	0.84	8.20	1.10	8.50	1.29
2	Papaverine	6.75	1.26	7.75	0.50	7.75	0.50	7.75	0.50	7.50	1.29
3	Magnesium sulfate	6.14	1.77	7.83	2.23	8.29	2.06	8.57	1.90	7.83	1.72
4	Control	8.50	1.87	8.60	2.07	8.67	1.86	8.50	1.64	8.50	1.64

**Table 2.** Mean venous external diameter measurements, in micrometers (µm) according to the time (t) evaluated, after drug administration.

Group	Drug	Time									
		t _0_		t _3_		t _5_		t _10_		t _30_	
		Mean	SD	Mean	SD	Mean	SD	Mean	SD	Mean	SD
1	Nitroglycerin	9.60	1.14	9.80	1.10	9.80	1.10	10.00	1.63	9.75	1.26
2	Papaverine	8.75	1.26	9.5	0.58	9.5	0.58	9.25	0.50	8.50	1.92
3	Magnesium sulfate	7.86	2.12	8.83	1.94	9.29	2.06	9.57	1.99	9.67	1.75
4	Control	9.17	1.47	8.40	1.67	8.83	1.84	9.50	1.76	9.83	1.94

 According to the measured values, the percentage of variation (Δ) in the diameter of the arterial and venous vessels was calculated in relation to the initial measurement at t _0_ (just before application of the drug) and the mean dilation value of each drug was calculated, used as a basis for statistical tests. 

After analyzing data homogeneity, a non-parametric Kruskal Wallis test was performed, between the different drugs and the control group, for the mean variation (Δ) in arterial external diameter according to the evolution of each group according to the time (Graph 1), observing no statistically significant difference in artery dilation values measured 3 minutes (p = 0.099), 5 minutes (p = 0.207) or 30 minutes (p = 0.183) after application of the drug. Statistically significant difference was observed for arterial dilation after 10 minutes with p=0.044.

**Figure 2.**
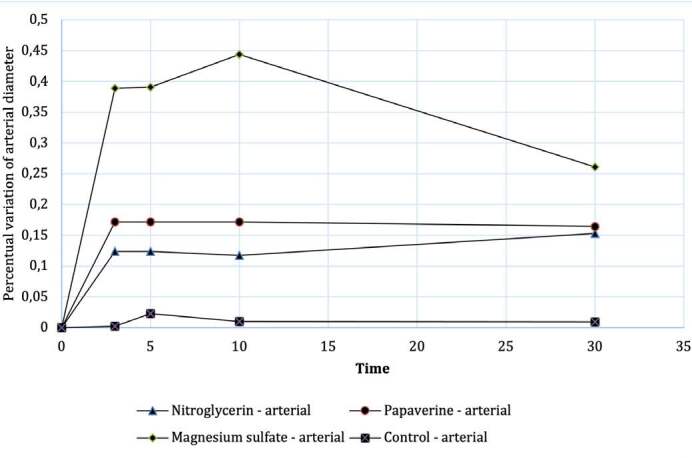
Evolution of the mean percentage change in arterial diameter as a function of time.

 In a comparative analysis between the different drugs and the control group, analyzing case by case, through the Mann-Whitney statistical test, it was observed that the only drug with a statistically significant increase in external diameter (vasodilation) was Magnesium sulfate when compared to the control group with p = 0.017. ( Figure 2 ) 

**Figure 3.**
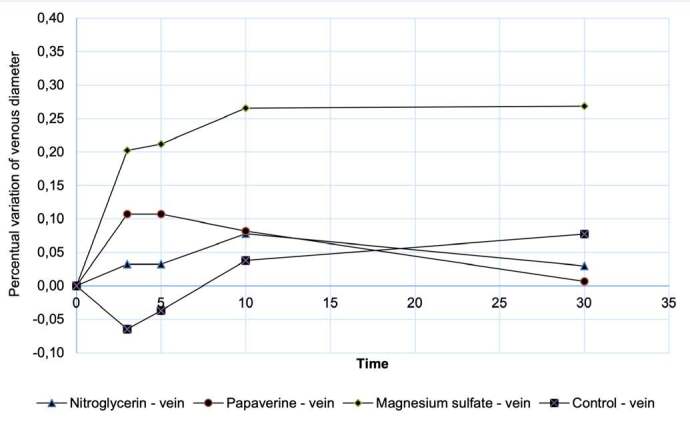
Evolution of the mean percentage change in venous diameter as a function of time.

 The analysis was repeated for the measurements of variation (Δ) in venous external diameter after topical application of the drugs and the control group, and no statistically relevant difference was observed in vein dilation values measured 3 minutes (p = 0.112), 5 minutes (p = 0.214), 10 minutes (p = 0.401) or 30 minutes (p = 0.243) after drug application. ( Figure 3 ) 

Magnesium Sulphate had a cost (FP) of R$ 219.07 per 200 ampoules (price per ampoule R$ 1.10), Papaverine R$ 177.45 per 10 ampoules (price per ampoule R$17.70) and Nitroglycerin R$ 364.71 per 10 ampoules (price per ampoule R$ 36.47)

## DISCUSSION

 This study was designed to assist in the choice of the most effective topical vasodilator drug in microsurgery, which is easily applicable at the intraoperative time and capable of controlling vasospasm, ischemia and thrombogenic factors at the intraoperative time of microanastomosis. ^16^ Vasodilation promotes an increase in the velocity of microanastomosis flow, better vascularization of the microsurgical flap and is correlated with a higher tissue survival rate. ^17^ Therefore, the hypothesis of this experimental study is that the greater and more prolonged the vasodilation caused by the drug, the greater the reduction of vasospasm and flow turbulence, as one of the factors of Virchow’s triad, consequent platelet activation with the endothelial surface and thrombus formation, ^18^ ^,^ ^19^ leading to an increased risk of vascular complications of the microsurgical flap. 

 When we observe, in our clinical practice, the impairment of flap perfusion parameters during the microsurgical procedure after the release of vascular clamps, one of the causative factors is arterial vasospasm with reduced flow to the transplanted tissue and the surgical tactic is the use of topical vasodilators, described in the medical literature and seen in the clinical practice of microsurgeons, with the objective of reducing the risk of anastomotic thrombosis and microcirculation immediately or late, with possible need for revision of the anastomoses, prolongation of the operative time, and increased complications. ^20^

 Although in the literature there are articles studying the options of vasodilating drugs for use in arterial vasospasm, there are no studies evaluating their effect on venous anastomosis, with venous thrombosis being one of the main complications observed in microsurgical flaps in limbs. ^21^ ^,^ ^22^ The comparative evaluation of venous vasodilation in our study did not demonstrate statistically relevant superiority of any of the drugs studied in relation to the control group, with no isolated influence on the success of venous microanastomosis. We emphasize that the main isolated clinical factor for success of microanastomosis is refined surgical technique. ^23^ We do not recommend the use of drugs to reduce venous vasospasm in our clinical practice. 

 In our study, the only drug that produced arterial vasodilation with a statistically significant result, when compared to the control group, was 10% Magnesium Sulfate, a drug used in cardiology, neurology and neurosurgery to treat vasospasm; however, its efficacy in microsurgery has not been confirmed. ^4^ ^,^ ^24^ In experimental microsurgery, magnesium sulfate is used as a drug with an antithrombotic effect , ^10^ its main side effects start at a plasma concentration of 7 Mg/dL-¹ and there may be electrocardiographic changes and, at higher plasma concentrations, areflexia, apnea and cardiac arrest, when above 24 Mg/dL-¹. Its use is safe at doses of 30 to 50 mg/kg intravenously, ^24^ ^-^ ^27^ therefore the use of 10% magnesium sulfate in reconstructive microsurgery with topical application of the drug is safe and has a serum concentration well below the safe dose by intravenous administration; however, in vivo studies should be performed to confirm its safety and efficacy in microanastomoses. 

 The choice of drugs and their corresponding doses was based on the best vasodilating outcomes of other studies ^4^ ^,^ ^13^ ^,^ ^16^ ^,^ ^28^ and on the availability of the drug in our setting. Magnesium sulfate is a drug widely available in Brazil’s Unified Health System and is included in the National List of Essential Medicines (RENAME 2022). ^29^ Papaverine or nitroglycerin are not included in RENAME 2022. 

 In our daily clinical practice, we routinely use papaverine as an arterial vasodilator after microanastomoses; however, this choice is based on personal choice and previous studies have not demonstrated consensus or level I clinical evidence for the use of vasodilators. Based on this lack of scientific basis to support the choice of the best arterial and venous vasodilating drugs ^7^ ^,^ ^8^ ^,^ ^9^ and the concern with public health care costs, we carried out this work with the inclusion of cost analysis of the drugs tested, all available in clinical-surgical practice. We observed that Magnesium Sulfate was the only drug that presented statistical superiority as an arterial vasodilator when compared to the control group and has an ampoule cost about 15 times lower than papaverine. ^14^ ^,^ ^15^ therefore, we suggest further experimental and clinical studies to confirm our results, which may provide sufficient evidence for review of the drug used as the gold standard for vasodilation in microsurgery. 

 Magnesium Sulfate may be a promising drug in reducing vasospasm in microsurgery, and may have superior effectiveness when compared to currently popular agents in use ^7^ ^,^ ^30^ being economically viable and widely available. ^29^

The limitations of this study include the costs of drugs and animals for experimental work, being randomized according to the availability for the work, being ideal the inclusion in future studies of a greater number of experimental tests.

## CONCLUSION

No drug studied exhibited a better topical vasodilating effect after venous microanastomosis. Magnesium sulfate showed a better arterial vasodilating effect when compared to the control group and the cost of this drug is the lowest among those tested in the Brazilian Health Care System. Further studies are needed to prove clinical evidence of the use of topical vasodilators for the reduction of intra- and postoperative complications of free flaps and microsurgical reconstruction in humans, induced by vasospasm and confirm what would be the gold standard drug for this purpose.
